# Donkey Milk Improves Dextran Sulfate Sodium‐Induced Ulcerative Colitis by Protecting Gut Barrier Function, Inhibiting the TLR4/MyD88/NF‐κB Signaling Pathway, and Modulating Gut Microbiota

**DOI:** 10.1002/fsn3.70989

**Published:** 2025-09-22

**Authors:** Lin Yang, Hua Ni, Xiaogang Gou, Bingqian Zhong, Keyi Wen, Yingying Zhang, Shicui Zhang, Yutao Wang

**Affiliations:** ^1^ Key Laboratory of Biological Resources and Ecology of Pamirs Plateau in Xinjiang Uygur Autonomous Region, College of Life and Geographic Sciences Kashi University Kashi China; ^2^ Xinjiang Yukunlun Natural Food Engineering Co., Ltd. Kashi China

**Keywords:** donkey milk, gut microbiota, intestinal barrier function, TLR4/MyD88/NF‐κB signaling pathway, ulcerative colitis

## Abstract

Donkey milk (DM) demonstrates promising bioactive properties, including anti‐inflammatory and microbiota‐modulating effects with therapeutic potential for ulcerative colitis (UC)—though mechanistic research remains limited. To bridge this research gap, a standardized DSS‐induced murine UC model was established. Results indicated that daily DM administration markedly attenuated clinical manifestations of UC, such as reduced body weight, intestinal mucosal injury, and increased Disease Activity Index (DAI). Mechanistically, DM enhanced gut barrier integrity through transcriptional upregulation of key genes (*MUC2*, *Reg3α*, *Reg3g*, *Occludin*, *Alpi*, *ZO‐1*) while suppressing inflammation via TLR4/MyD88/NF‐κB cascade inhibition and concomitant reduction of proinflammatory cytokines (TNF‐α, IL‐1β, IL‐18). Cecal 16S rDNA sequencing further confirmed DM‐mediated microbial restoration, characterized by significant enrichment of beneficial genera (*Enterorhabdus*, *Lachnoclostridium*, *Parvibacter*) and reduction of inflammation‐associated taxa (*Bacteroides*, *norank_Muribaculaceae*, *Eubacterium fissicatena group*). In conclusion, our integrated analysis validates DM as a nutraceutical intervention capable of simultaneously targeting mucosal healing, inflammation resolution, and microbiome restoration—positioning it as a promising therapeutic and prophylactic approach for IBD spectrum disorders.

## Graphical abstract

This study confirms that Donkey milk (DM) intervention significantly alleviates clinical symptoms of dextran sulfate sodium (DSS)‐induced ulcerative colitis (UC) in mice, including weight loss, colonic tissue damage, and elevated DAI scores. Mechanistic investigations demonstrate that DM effectively promotes intestinal damage repair by suppressing inflammatory responses and maintaining intestinal barrier integrity. Further mechanistic analysis suggests that DM likely promotes intestinal repair in UC by inhibiting the TLR4/MyD88/NF‐κB signaling pathway and ameliorating gut microbiota dysbiosis. Collectively, as a nutritional intervention, DM achieves three key objectives simultaneously: mucosal repair, inflammation resolution, and restoration of microbiome homeostasis. This positions DM as a promising preventive and therapeutic strategy for UC and related inflammatory bowel diseases.

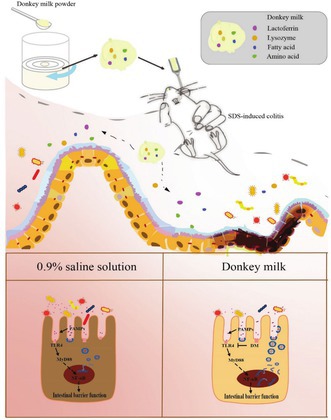

## 1 Introduction

Ulcerative colitis (UC), characterized by chronic relapsing inflammation of the colonic mucosa, arises from dysregulated immune responses in genetically predisposed individuals. Core clinical features include bloody diarrhea and weight loss. Left untreated, most patients experience progressive disease courses culminating in complications such as toxic megacolon or colitis‐associated cancer (Le Berre et al. 2023; Wangchuk et al. 2024). It is estimated that approximately 5 million people worldwide will suffer from UC by 2030, making it a significant global healthcare challenge (Le Berre et al. 2023). UC consequently constitutes a priority therapeutic area in digestive health. Despite advances, UC persists as an incurable condition where current interventions merely maintain remission. First‐line agents (5‐ASAs, corticosteroids, thiopurines) incur substantial safety concerns: 5‐ASAs associate with interstitial nephritis, corticosteroids induce metabolic complications and dependency, while immunomodulators increase lymphoma risk (Feuerstein et al. 2019). The therapeutic paradigm centers on endoscopic mucosal healing induction and durable deep remission maintenance, critical to translating to significant reductions in hospitalization frequency and colectomy probability (Wangchuk et al. 2024). Given these limitations, there is an urgent imperative to develop alternative therapeutic agents that simultaneously offer superior safety margins and enhanced efficacy profiles to optimize clinical management strategies for UC.

Chronic mucosal inflammation characterizes UC pathogenesis, driven by interdependent mechanisms: (1) compromised epithelial barrier function, (2) dysregulated immune responses, and (3) dysbiosis with reduced microbial diversity (Le Berre et al. 2023). The precise hierarchical interplay between these components continues to challenge researchers. Colonic tissues from UC patients demonstrate elevated IL‐1β, IL‐6, and TNF‐α expression versus healthy controls (Voshagh et al. 2024). These cytokines disrupt epithelial tight junctions (ZO‐1↓, occludin↓), enabling bacterial translocation into the lamina propria (X. Zhang et al. 2023). This initiates a pathogenic cascade amplified by characteristic dysbiosis, particularly Proteobacteria enrichment observed in UC patients—which sustains NF‐κB‐mediated chronic inflammation through persistent Microbe‐Associated Molecular Patterns (MAMPs) signaling (X. Chen et al. 2020; Čipčić Paljetak et al. 2022; Ge et al. 2020; Imhann et al. 2018; Zhu et al. 2022). Consequently, controlling inflammation and restoring microbial homeostasis have emerged as promising therapeutic strategies for UC management.

Donkey milk (DM) represents a nutritionally dense dairy matrix with favorable lipidomics (low saturated fat/cholesterol; high MUFA/PUFA content) and bioactive enrichment (lysozyme, lactoferrin, vitamin D₃) (L. Li et al. 2017; Živkov Baloš et al. 2023). Its compositional homology with human milk underlies exceptional bioavailability. DM's documented bioactivities, including bacteriostatic effects against enteropathogens, TNF‐α suppression, and glutathione peroxidase enhancement, support its nutraceutical potential for inflammatory disorders (Martini et al. 2021). Accumulating evidence supports DM's therapeutic efficacy against gastric ulcers, respiratory comorbidities in metabolic disorders, and type 2 diabetes management (Garhwal et al. 2022; Yan Li et al. 2020; Sami et al. 2025). Population studies further indicate that consistent dairy intake correlates with lower IBD risk (Almofarreh et al. 2024). Furthermore, DM administration attenuates chronic stress‐induced intestinal hyperpermeability and mitigates inflammation‐mediated intestinal injury (Yvon et al. 2019). These findings indicate DM possesses promising therapeutic potential for UC. Given the nascent stage of mechanistic research, we utilized DSS‐induced colitis in C57BL/6 mice to systematically evaluate: (1) DM's disease‐modifying effects (Disease Activity Index [DAI], histology, colon length), (2) mucosal barrier restoration (ZO‐1, occludin, MUC2 expression), (3) TLR4/NF‐κB pathway modulation, and (4) microbiota restructuring (16S rRNA sequencing). This integrated approach provides scientific evidence supporting DM's clinical application potential while advancing mechanistic understanding of its pharmacological activity.

## 2 Materials and Methods

### 2.1 Materials and Reagents

The sources of the experimental reagents were as follows: Dextran sulfate sodium (DSS, 36–50 KDa), MP Biomedicals (Shanghai, China); DM powder, Yukunlun Natural Food (Kashi, China); Stool occult blood test kit, Leagene (Beijing, China); Mouse ELISA kits (TNF‐α, IL‐1β, and IL‐18), Meimian (Wuhan, China); RNApure Fast Tissue and Cell Kit, Kongshibo (Taizhou, China); HiScript II Q RT SuperMix for qPCR, Vazyme (Nanjing, China); GoTaq qPCR Master Mix, Promega (Beijing, China); Full protein extraction kit, Solarbio (Beijing, China); Modified BCA Protein Assay Kit, Sangon Biotech (Shanghai, China); and Primary antibodies (TLR4, MYD88, p65, p‐p65, and GAPDH) and HRP‐conjugated rabbit and mouse secondary antibodies, Proteintech (Nanjing, China).

### 2.2 Experimental Animals

Forty 6‐8‐week‐old male C57BL/6 mice (Jiangsu Huachuang Xinnuo Pharmaceutical Technology Co. Ltd.) were housed in SPF facilities with controlled conditions (22°C ± 1°C, 55% ± 5% humidity, 12/12‐h light/dark cycle). Animals received irradiated feed and autoclaved water ad libitum, with twice‐weekly bedding replacement. All experimental procedures complied with the NIH Guide for the Care and Use of Laboratory Animals (8th edition, 2011) and were approved by the institutional IACUC (SY‐2024‐05‐15001; May 15, 2024).

### 2.3 Induction and Intervention of UC

Following a 7‐day acclimation period, mice were randomly assigned to four groups (10 mice per group): normal control (NC), donkey milk control (DMC), UC model (DSS), and donkey milk treatment (DM_DSS). NC and DM_DSS groups received daily oral gavage of DM (0.1 g/mL) for 21 days, while control groups were given equivalent volumes of physiological saline. UC was induced in DSS and DM_DSS groups by administering 3% DSS in drinking water ad libitum from days 15–21, per established protocols (Wirtz et al. 2017). Mortality occurring during the study led to the exclusion of subjects (NC: *n* = 2; DMC: *n* = 2; DSS: *n* = 3; DM_DSS: *n* = 1). Surviving mice underwent 12‐h fasting before terminal blood collection from the submandibular venous plexus. Serum aliquots were obtained following centrifugation (4000 *g*, 10 min) and cryopreserved at −80°C. After cervical dislocation euthanasia, colon length was measured. Distal colon segments (1–2 cm) were formalin‐fixed (10%, 48 h), with residual colonic tissue and cecal contents flash‐frozen at −80°C. The gavage dosage of 0.9 mL/d for each mouse was calculated according to Formula (1) (Wei et al. 2011).

(1) Doseb=Dosea×kbka×WbWa23

where Dose_(*b*)_ represents gavage volume per mouse (mL/day); Dose_(*a*)_ is the recommended daily dairy intake for adults (300 mL) per the Chinese Nutrition Society (2022); *k*_*a*_ and *k*_*b*_ are the allometric scaling factors for humans and mice, respectively (*k*_*a*_ = 0.1, *k*_*b*_ = 0.06); *W*_*a*_ and *W*_*b*_ indicate the mean body weights of adult humans and mice (*W*_*a*_ = 60 kg, *W*_*b*_ = 0.02 kg).

### 2.4 DAI and Histological Analysis

Body weight changes, stool consistency, and gross fecal blood were monitored daily. DAI scores were calculated according to established criteria (Table 1) following standardized methods (S. Wu, Wu, and Chen 2023). The final DAI value represented the arithmetic mean of combined parameter scores.

**TABLE 1:** Scoring criteria of DAI.

Stool consistency for diarrhea	Fecal occult blood	Weight loss (%)	Score
Normal	−	0–1	0
Soft but still formed	+	1–5	1
Soft	++	5–10	2
Soft and wet	+++	10–20	3
Watery diarrhea	++++	≥ 20	4

Distal colonic specimens were fixed in 10% formalin, dehydrated through a graded ethanol series, and paraffin‐embedded. Tissue sections (5 μm thick) were cut using a microtome and stained with hematoxylin and eosin (H&E). Assessment of histological alterations utilized a standardized scoring criterion (Table 2) as documented in previous literature (Koelink et al. 2018).

**TABLE 2:** Histological score criteria.

Inflammatory infiltrate	Goblet cell loss	Crypt damage	Mucosal damage	Score
None	None	None	None	0
Increased presence of inflammatory cells	< 10%	Loss of basal 1/3 of crypt	Mucus layer	1
Infiltrates also in submucosa	10%–50%	Loss of basal 2/3 of crypt	Submucosa	2
Transmural	> 50%	Loss of entire crypt	Muscular and serosa	3
		Crypt and surface epithelium lost		4

### 2.5 Measurement of Inflammatory Cytokines in Colonic Tissue

ELISA kits were used to quantify the levels of IL‐1β, TNF‐α, and IL‐18 in colonic tissues.

### 2.6 RT‐qPCR Analysis

Colonic tissue RNA was extracted with RNApure Fast Tissue and Cell Kit and quality‐verified by spectrophotometry (Allsheng, Hangzhou, China). cDNA was synthesized from RNA using RT SuperMix. Quantitative PCR used BRYT Green dye chemistry on a 7500 Fast System (Applied Biosystems, USA). GAPDH was used as the internal reference control for normalization. Gene expression levels were calculated using the 2^−ΔΔ*Ct*^ method, with primer sequences listed in Table 3.

**TABLE 3:** The information on the primers.

Gene name		Primer
GAPDH	Forward	5′‐CATCACTGCCACCCAGAAGACTG‐3′
	Reverse	5′‐ATGCCAGTGAGCTTCCCGTTCAG‐3′
TLR4	Forward	5′‐CAGAGCCGTTGGTGTATC‐3′
	Reverse	5′‐AGTTGCCGTTTCTTGTTC‐3′
MyD88	Forward	5′‐AGGACAAACGCCGGAACTTTT‐3′
	Reverse	5′‐GCCGATAGTCTGTCTGTTCTAGT‐3′
NF‐κB p65	Forward	5′‐AAATGGGAAACCGTATGAGCCTGTG‐3′
	Reverse	5′‐GTTGTAGCCTCGTGTCTTCTGTCAG‐3′
ZO‐1	Forward	5′‐GTTGGTACGGTGCCCTGAAAGA‐3′
	Reverse	5′‐GCTGACAGGTAGGACAGACGAT‐3′
Occludin	Forward	5′‐TGGCAAGCGATCATACCCAGAG‐3′
	Reverse	5′‐CTGCCTGAAGTCATCCACACTC‐3′
MUC2	Forward	5′‐GCTGACGAGTGGTTGGTGAATG‐3′
	Reverse	5′‐GATGAGGTGGCAGACAGGAGAC‐3′
Reg3α	Forward	5′‐ATCGCTCCCACTGCTATGCC‐3′
	Reverse	5′‐TCCACTCTGCCGTTCACCAA‐3′
Reg3γ	Forward	5′‐CATCCACCTCTGTTGGGTTC‐3′
	Reverse	5′‐TTCCTGTCCTCCATGATCAAA‐3′
ALPi	Forward	5′‐GGCTACACACTTAGGGGGACCTCCA‐3′
	Reverse	5′‐AGCTTCGGTGACATTGGGCCGGTT‐3′

### 2.7 Western Blot Analysis

Total proteins were extracted from colonic tissues with a commercial protein extraction kit according to the manufacturer's protocol. Protein concentrations were subsequently quantified using a BCA assay kit. Proteins (equal loading) were resolved on 10%–12% SDS‐PAGE gels and electroblotted to nitrocellulose membranes (Cytiva, Shanghai, China). After blocking membranes with 5% non‐fat milk for 2 h and washing thrice with TBST, the membranes were incubated overnight at 4°C with primary antibodies against: GAPDH (1:50,000), TLR4 (1:1000), MyD88 (1:2000), p65 (1:1000), and p‐p65 (1:1000). After three TBST washes, membranes were probed with HRP‐secondary antibodies (1 h), then developed with ECL substrate (Tanon, Shanghai, China). Protein bands were visualized using the Gel Doc XR+ system (Bio‐Rad, Hercules, USA) and quantified by densitometry with ImageJ v1.54f (NIH, Bethesda, USA).

### 2.8 Gut Microbiota Analysis

Total DNA was extracted from cecal content samples. The V3–V4 hypervariable region of the bacterial 16S rRNA gene was amplified via PCR using primers 341F (5′‐CCTACGGGNGGCWGCAG‐3′) and 805R (5′‐GACTACHVGGGTATCTAATCC‐3′). Purified amplicons were prepared into sequencing libraries and subjected to high‐throughput sequencing on an Illumina platform. Gut microbiota sequencing and bioinformatic analyses were conducted by Sangon Biotech (Shanghai, China).

### 2.9 Statistical Analysis

Data are presented as mean ± SEM from at least three independent biological replicates. Statistical analyses used GraphPad Prism 9.5 (GraphPad Software, San Diego, USA) and SIMCA 14.1 (Umetrics, Umea, Sweden). Intergroup comparisons used unpaired *t*‐tests (two groups) or one‐way ANOVA with post hoc tests (multiple groups), with statistical significance defined as *p* < 0.05 for all analyses.

## 3 Results

### 3.1 DM Alleviates Clinical Symptoms in UC Mice

**FIGURE 1:**
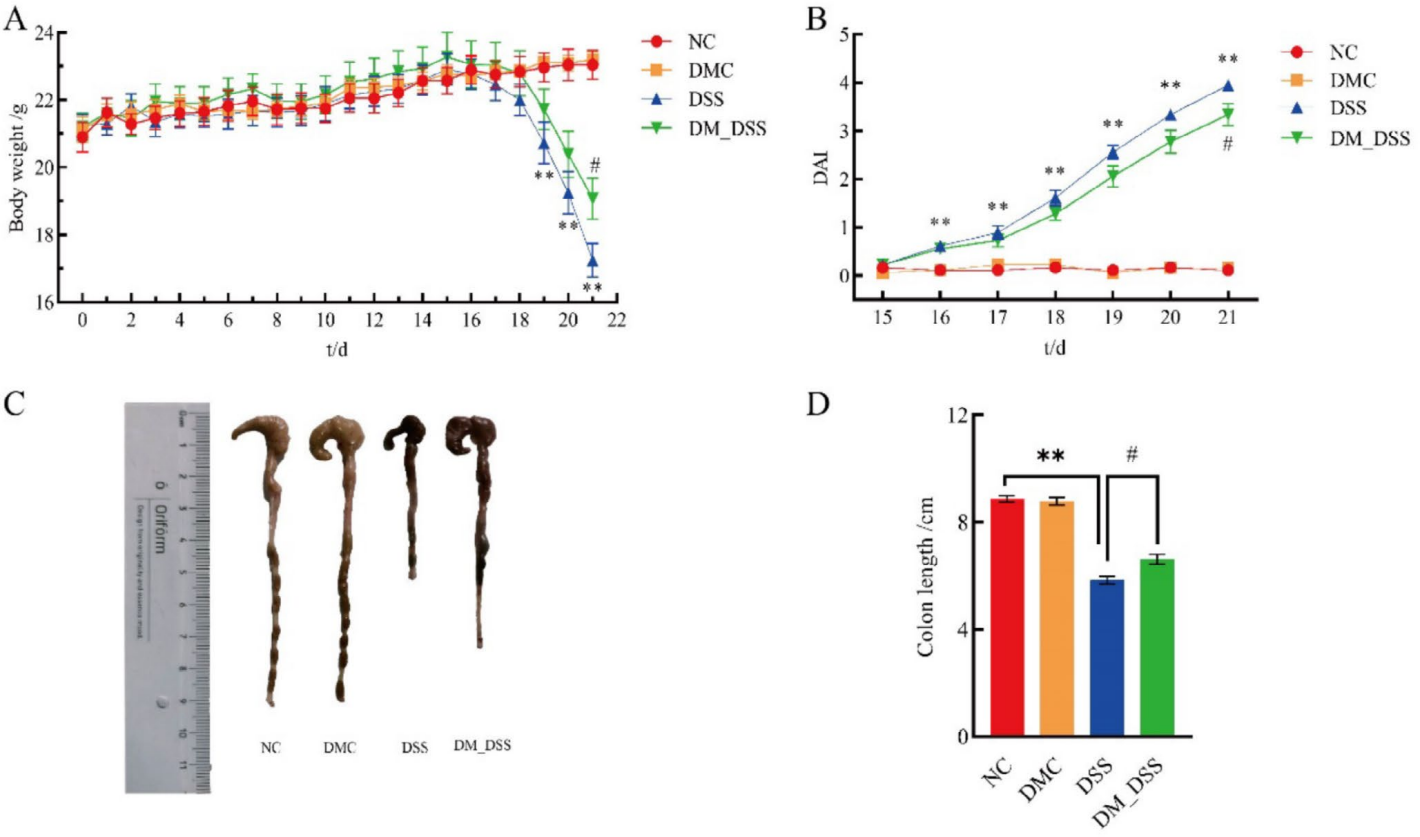
DM relieves DSS‐induced ulcerative colitis in mice. (A) Body weight, *n* ≥ 7; (B) Disease Activity Index (DAI) score, *n* = 6; (C) representative photographs of the colon, *n* ≥ 7; (D) colon length, *n* ≥ 7. The values are expressed as mean ± SEM **p < 0.01 versus NC group; ^#^ *p* < 0.05 versus DSS group.

Pre‐induction weight curves confirmed cohort homogeneity (*p* > 0.05). Acute weight loss occurred in DSS‐exposed groups by day 4 (*p* < 0.01). At endpoint (day 21), DM_DSS mice retained significantly greater mass than DSS controls (*p* < 0.05) (Figure 1A). Pathological colon shortening in colitis reflects inflammation‐driven and bacterial complication‐driven mucosal injury (Guo et al. 2024). Colitis progression was quantified via DAI (weight loss + stool consistency + fecal occult blood). DSS mice showed severe disease manifestation: significantly elevated DAI scores and colon shortening versus NC controls (*p* < 0.01). DM intervention substantially ameliorated both DAI scores and colon shortening (*p* < 0.05) (Figure 1B–D). These results suggest that DM intervention exerts therapeutic effects on UC.

### 3.2 DM Attenuates Histopathological Damage in UC Mice

**FIGURE 2:**
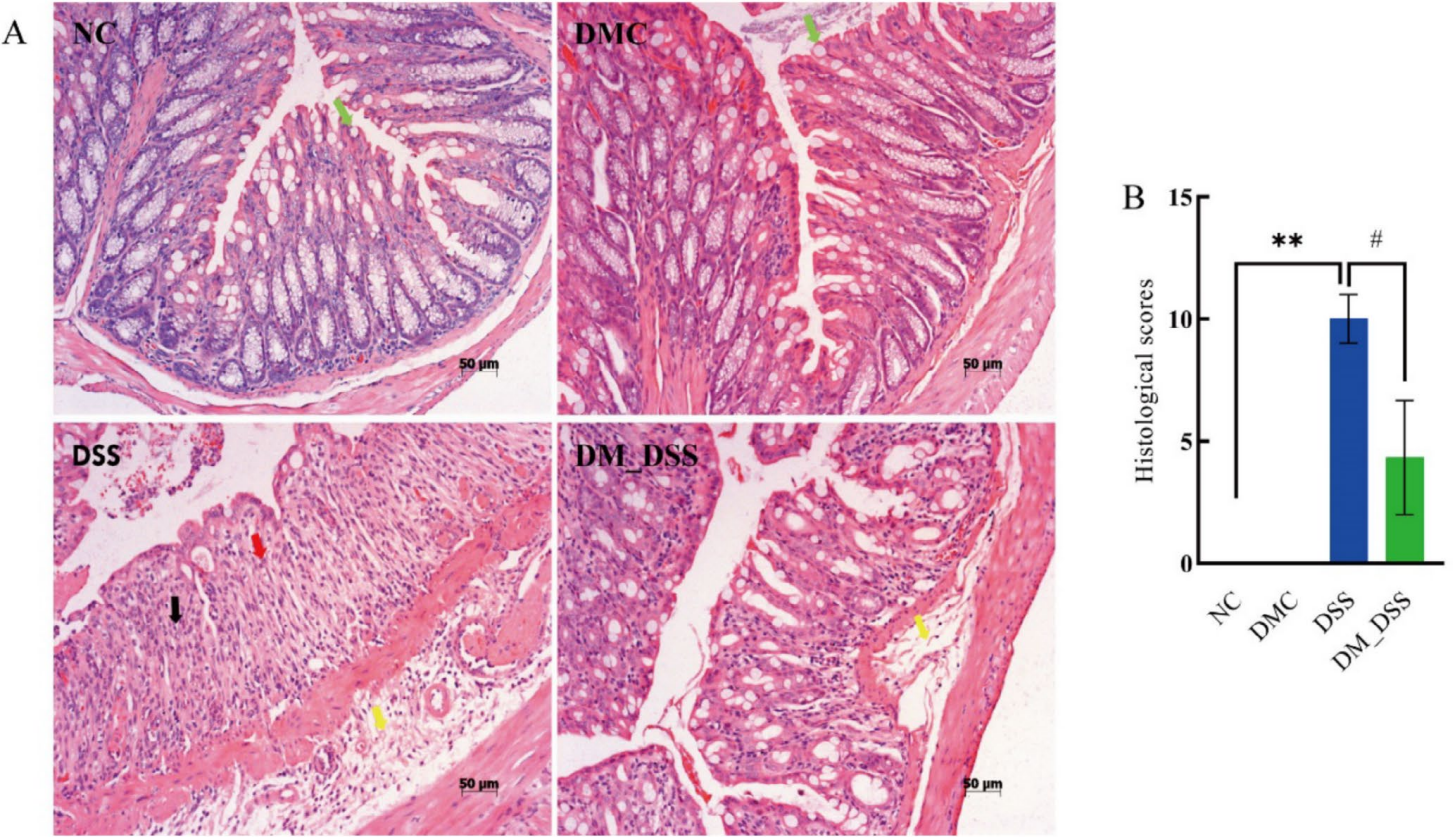
Histopathology of mouse colitis under treatment. (A) Pathological sections of colonic tissues revealed by HE staining (scale bars: 50 μm); (B) Histological scores were evaluated. The green arrow indicates goblet cells; the yellow arrow indicates inflammatory cell infiltrates; the black arrow represents proliferating stromal cells (e.g., fibroblasts); the red arrow indicates areas of epithelial denudation in mucosal layer. The values are expressed as mean ± SEM (*n* = 3) **p < 0.01 versus NC group; ^#^ *p* < 0.05 versus DSS group.

Histopathological analysis employing H&E staining assessed DM's protective efficacy against intestinal tissue damage in experimental colitis. Histopathological analysis revealed intact colonic architecture with normal crypt morphology and absence of inflammatory infiltrates in both NC and DMC groups. In contrast, DSS‐treated mice exhibited extensive mucosal ulceration with complete crypt loss and marked inflammatory cell infiltration. Compared to DSS‐treated mice, DM intervention significantly attenuated colonic mucosal lesions and suppressed inflammatory cell infiltration (Figure 2A). DM intervention significantly reduced histopathological severity scores in UC mice compared to DSS controls (*p* < 0.05), as assessed by standardized metrics for crypt damage and inflammation (Figure 2B). Data conclusively establish DM's therapeutic efficacy against intestinal inflammation and structural damage in experimental UC.

### 3.3 DM Improves Intestinal Barrier Function in UC Mice

**FIGURE 3:**
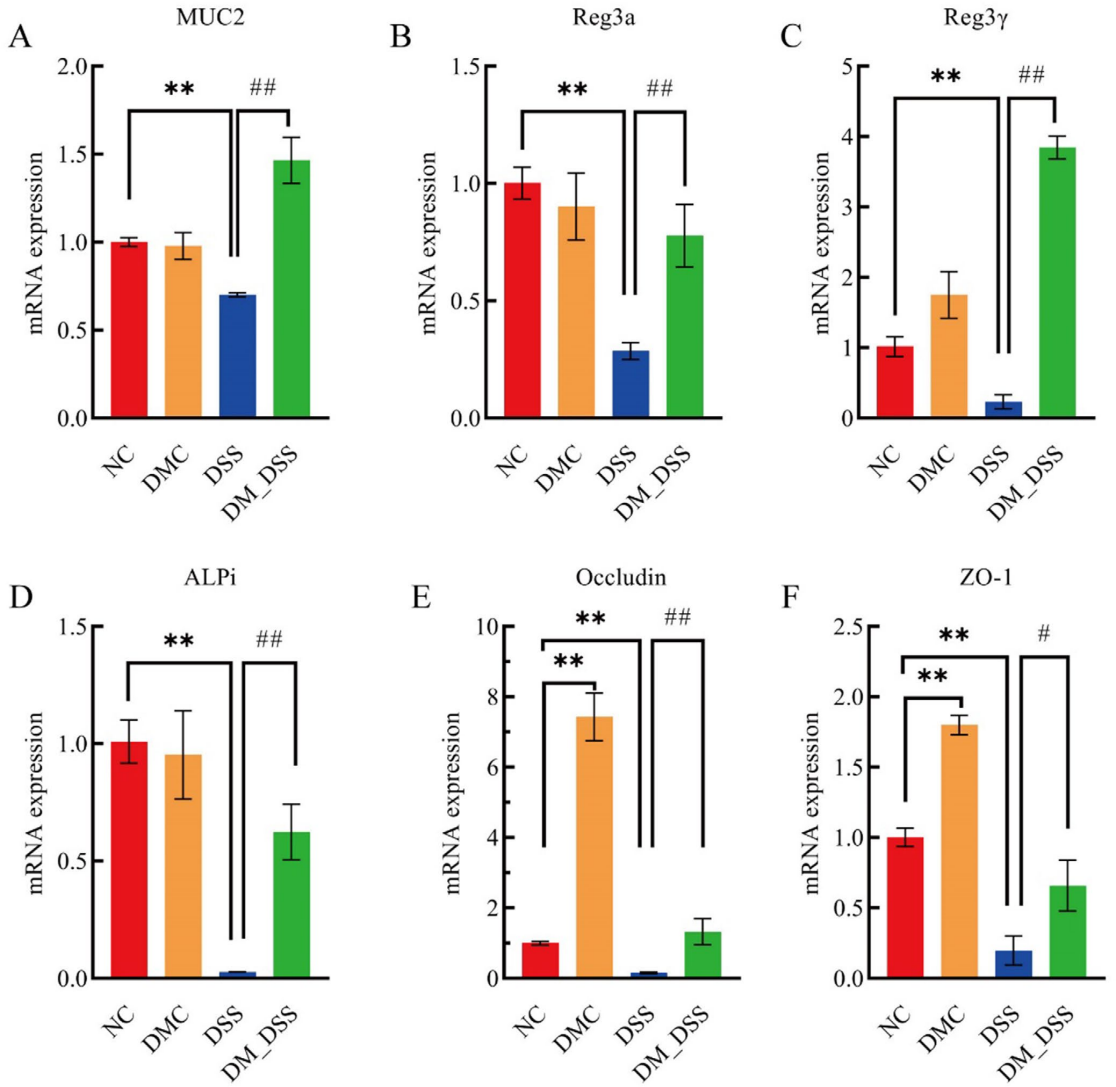
Colonic barrier gene expression: (A) MUC2, (B) Reg3α, (C) Reg3g, (D) Alpi, (E) Occludin, and (F) ZO‐1. The values are expressed as mean ± SEM (*n* = 3). **p < 0.01 versus NC group; ^#^ *p* < 0.05, ^##^ *p* < 0.01 versus DSS group.

As guardian of mucosal homeostasis, the intestinal barrier orchestrates commensal‐immune equilibrium while providing frontline defense against luminal threats (Stolfi et al. 2022). This mucin scaffold creates essential biophysical compartmentalization between epithelium and luminal environment, with embedded defense molecules (intestinal alkaline phosphatase, antimicrobial peptides) executing direct pathogen neutralization (Achasova et al. 2021; Gubatan et al. 2021; Singh and Lin 2021). Additionally, defects in tight junction proteins (e.g., ZO‐1, Claudin‐1) directly compromise epithelial barrier integrity, enabling enhanced microbial invasion (Chelakkot et al. 2018). Compared to normal controls, DSS exposure markedly suppressed transcription of key barrier genes: mucin‐encoding MUC2, antimicrobial lectins Reg3α/γ, phosphatase Alpi, and tight junction components ZO‐1 and Occludin (*p* < 0.05). DM treatment rescued this transcriptional deficit (*p* < 0.05) (Figure 3E,F). Collectively, DM administration promoted the transcriptional activation of intestinal barrier function genes, implying its capacity to counteract intestinal dysfunction during inflammatory exacerbation.

### 3.4 DM Inhibits Colonic Inflammatory Cytokine Levels and the TLR4/MyD88/NF‐κB Signaling Pathway in UC Mice

**FIGURE 4:**
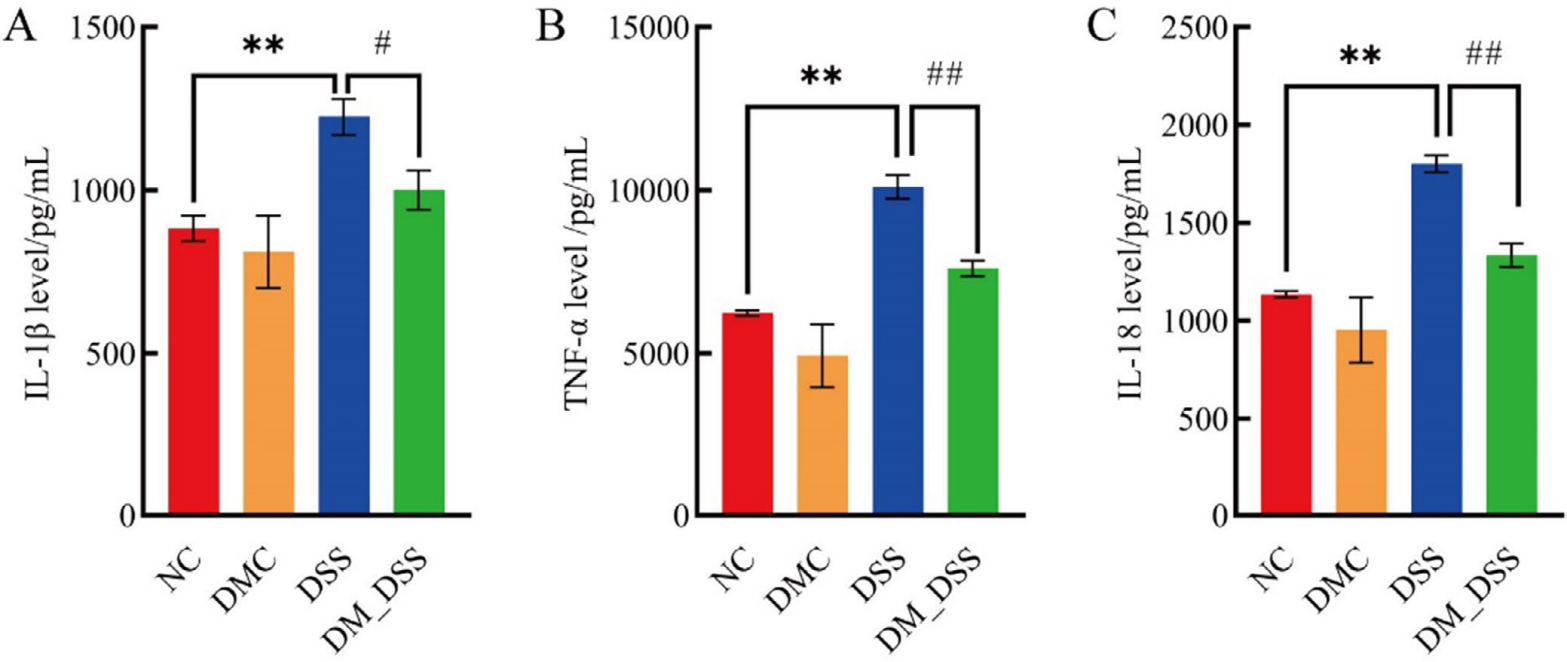
Alterations in proinflammatory cytokine expression in the colon: (A) IL‐1β, (B) TNF‐α, and (C) interleukin 18 (IL‐18). The values are expressed as mean ± SEM (*n* = 6) **p < 0.01 versus NC group; ^#^ *p* < 0.05, ^##^ *p* < 0.01 versus DSS group.

**FIGURE 5:**
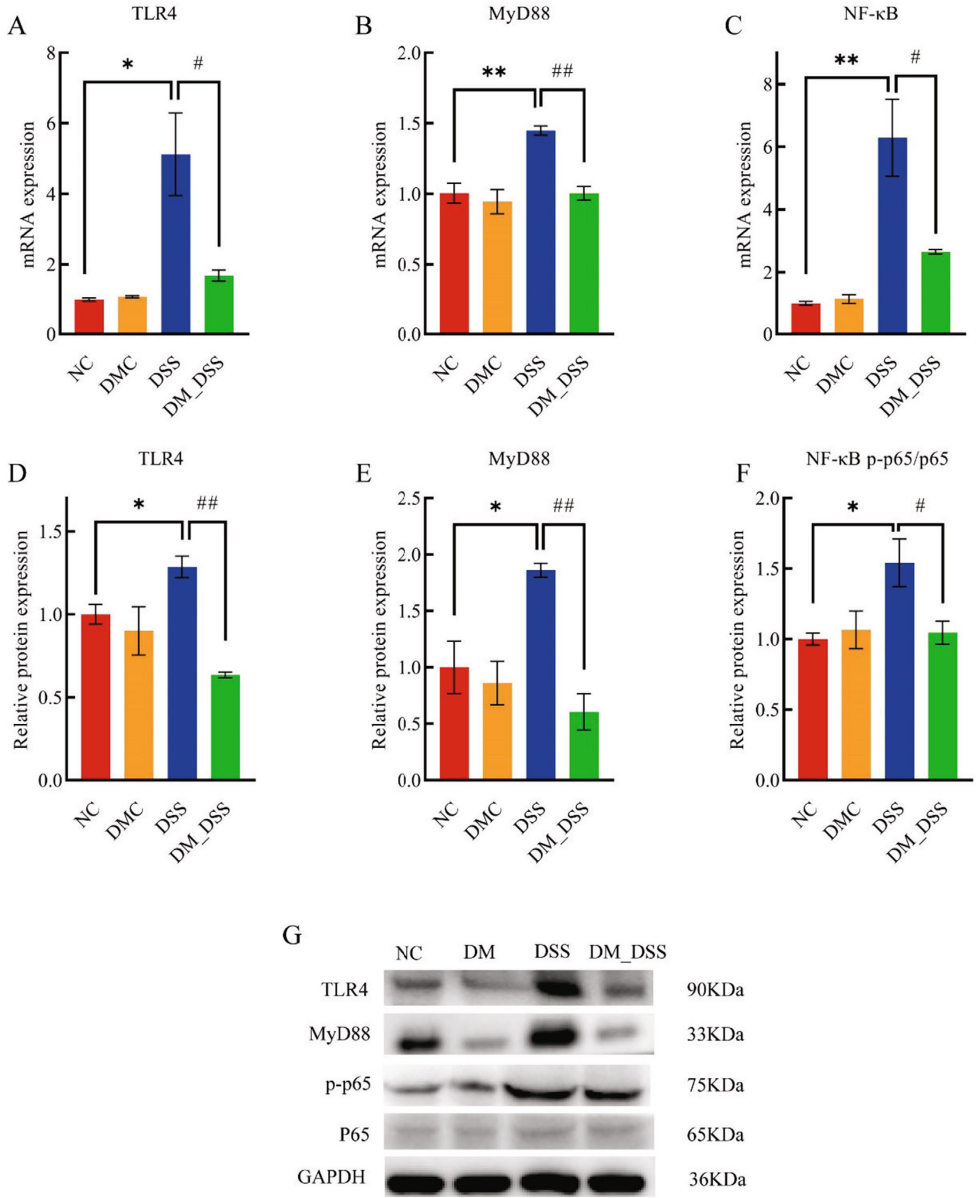
TLR4/MyD88/NF‐κB pathway analysis in colonic tissues. (A–C) Gene expression; (D–G) protein levels. The values are expressed as mean ± SEM (*n* = 3) *p < 0.05, **p < 0.01 versus NC group; ^#^ *p* < 0.05, ^##^ *p* < 0.01 versus DSS group.

Pathological elevation of IL‐1β and TNF‐α correlates with UC severity progression (Voshagh et al. 2024). To determine DM's local anti‐inflammatory efficacy, we quantified key pro‐inflammatory cytokines (TNF‐α, IL‐1β, IL‐18) in colonic tissues. Colonic tissues from DSS‐induced colitis models exhibited significantly increased TNF‐α, IL‐1β, and IL‐18 levels relative to healthy controls (*p* < 0.01). Therapeutic administration of DM markedly suppressed these proinflammatory cytokines (*p* < 0.05, Figure 4). To elucidate the mechanism underlying DM's suppression of pro‐inflammatory cytokines, this study investigated its impact on the TLR4/MyD88/NF‐κB pathway. Notably, compared with normal controls, mice in the UC group exhibited significantly elevated expression of TLR4 and MyD88 proteins alongside enhanced NF‐κB phosphorylation in colonic tissues (*p* < 0.05). This indicates that DSS‐induced colitis activates the TLR4/MyD88/NF‐κB signaling cascade. Furthermore, DM intervention markedly reduced TLR4/MyD88 protein abundance and NF‐κB phosphorylation (*p* < 0.05), confirming DM's inhibitory effect on TLR4/MyD88/NF‐κB pathway activation in DSS‐induced colitis (Figure 5A–G). In summary, the therapeutic potential of DM in UC involves blocking TLR4‐dependent NF‐κB activation, thereby reducing colonic tissue levels of TNF‐α, IL‐1β, and IL‐18.

### 3.5 DM Modulates the Gut Microbiota in UC Mice

**FIGURE 6:**
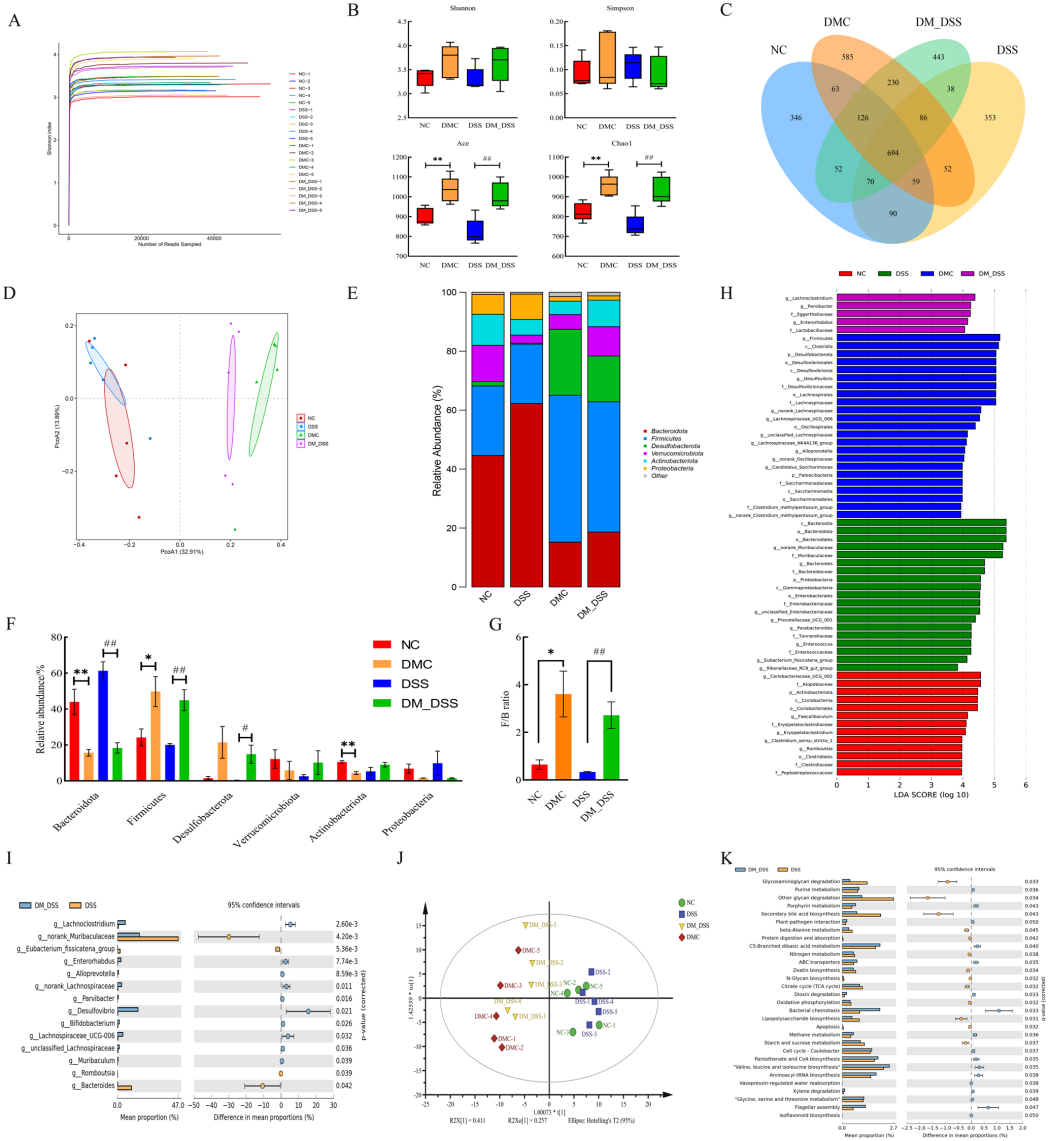
Effect of DM on the regulation of the gut microbiota in cecum content. (A) Shannon index curve; (B) alpha diversity assessment using richness (Chao1, Ace) and evenness (Shannon, Simpson) indices; (C) gut microbiota β‐diversity: PCoA ordination; (D) Venn diagram; (E) phylum‐level relative abundance bar plot; (F) differentially abundant phyla; (G) *Firmicutes/Bacteroidetes* (F/B) ratio; (H) linear discriminant analysis effect size (LEfSe) results (LDA > 2); (I) STAMP analysis of differentially abundant genera (DSS vs. DM_DSS); (J) OPLS‐DA of KEGG L3 functional profiles across groups; (K) differentially abundant KEGG L3 pathways: DSS versus DM_DSS (STAMP). The values are expressed as mean ± SEM (*n* = 5) *p < 0.05, **p < 0.01 versus NC group; ^#^ *p* < 0.05, ^##^ *p* < 0.01 versus DSS group.

As sequencing depth increased, Shannon index curves plateaued, indicating adequate capture of microbial diversity in the dataset (Figure 6A). Although microbial diversity was comparable between the DSS group and normal controls (NC), DM treatment significantly enhanced microbial richness (Figure 6B). Venn diagram analysis revealed that the DMC group harbored the highest number of unique OTUs (585), followed by the DM_DSS group (443) (Figure 6C). Principal Coordinates Analysis (PCoA) showed separation trends in gut microbiota structure between the DM_DSS group and the NC, DSS, or DMC groups (Figure 6D). At the phylum level, DM treatment significantly modulated the abundance of Bacteroidota, Firmicutes, and Desulfobacterota in fecal microbiota compared to the DSS group, resulting in a marked increase in the Firmicutes/Bacteroidota (F/B) ratio (Figure 6E–G). Linear discriminant analysis effect size (LEfSe) analysis identified differentially abundant bacterial taxa associated with DM treatment. LDA distribution analysis revealed significant enrichment of *Bacteroides*, *norank_Muribaculaceae*, *Prevotellaceae_UCG_001*, and *Eubacterium_fissicatena_group* in the DSS group. Furthermore, *Lachnoclostridium*, *Parvibacter*, and *Enterorhabdus* exhibited significant enrichment in the DM_DSS group (Figure 6H). Contrasted with DSS, DM intervention downregulated *Bacteroides*, *norank_Muribaculaceae*, and *Eubacterium_fissicatena_group* while upregulating *Lachnoclostridium*, *Parvibacter*, *Enterorhabdus*, and *Desulfovibrio* (Figure 6I). Based on 16S rRNA sequencing of microbial community profiles, PICRUSt analysis was employed to investigate differences in KEGG pathways across four gut microbiota groups. Orthogonal partial least squares‐discriminant analysis (OPLS‐DA) demonstrated significant intergroup separations (Figure 6J). Between DSS and DM_DSS groups, notable alterations in host metabolic functions were observed pertaining to Metabolism of cofactors and vitamins, Glycan biosynthesis and metabolism, Lipid metabolism, Xenobiotics biodegradation and metabolism, and Amino acid metabolism. Significant decreases in functional abundance were observed for 13 KEGG pathways in the DM‐treated group versus the DSS group, encompassing: Glycan biosynthesis and metabolism (Glycosaminoglycan degradation; Other glycan degradation; N‐Glycan biosynthesis) and Lipid metabolism (Secondary bile acid biosynthesis); conversely, 17 others showed significant upregulation, including Metabolism of cofactors and vitamins (Porphyrin metabolism; Pantothenate and CoA biosynthesis) and Amino acid metabolism (Glycine, serine, and threonine metabolism; Valine, leucine, and isoleucine biosynthesis) (Figure 6K). Collectively, these findings indicate that DM ameliorates UC through modulation of the gut microbiota.

## 4 Discussion

Natural products show efficacy against inflammatory diseases, yet research on the impact of DM on UC remains limited (Yaxi Zhou, Wang, and Yan 2023). This study confirmed that DM treatment alleviates adverse symptoms of DSS‐induced UC in mice, including body weight loss and colon damage, and increased DAI score. The treatment of DM effectively promoted the repair of intestinal injury by inhibiting the inflammation and preserving the integrity of the intestinal barrier function. Mechanistically, DM might promote intestinal repair in UC by suppressing the TLR4/MyD88/NF‐κB signaling pathway and ameliorating gut dysbiosis. Collectively, these results establish DM as a promising therapeutic candidate for UC.

Functioning as a dynamic selective barrier, the intestinal epithelium concurrently enables nutrient/electrolyte absorption and prevents pathogen/antigen invasion, thereby maintaining mucosal immune homeostasis. Its dysfunction represents a critical player in both the initiation and progression of UC. The absence of the mucin2 (MUC2) will lead to chemical barrier disruption, thereby inducing the initiation and progression of colitis (Johansson et al. 2008). Deficient antimicrobial secretion (Reg3α/γ, ALPI) from gut epithelial/Paneth cells initiates dysbiosis‐driven inflammation that disrupts barrier integrity (Gubatan et al. 2021; Liu et al. 2016). Moreover, disruption of intercellular tight junctions compromises the intestinal mechanical barrier, increasing permeability and exacerbating colonic tissue damage (Chelakkot et al. 2018; Yuqing Wu et al. 2022). Studies confirm markedly reduced expression of tight junction proteins ZO‐1 and Claudin‐1 in the colonic mucosa of UC patients (Kucharzik et al. 2001; Kuo et al. 2021). Emerging evidence suggests ZO‐1 may additionally mediate mucosal repair processes in UC patients (Kuo et al. 2021). Our study demonstrated similar findings that DSS caused intestinal barrier dysfunction in mice, including downregulated expression of genes such as *ZO‐1*, *occludin*, and *MUC2*, and DM treatment significantly reversed this trend. Numerous in vivo studies have shown that organic macromolecules from plants and animals affect intestinal barrier function. For example, dietary tryptophan supplementation or exogenous lysozyme administration can strengthen intestinal barrier integrity through transcriptional activation of tight junction proteins (e.g., ZO‐1/Occludin) (Liang et al. 2018; Yuying Wu, Cheng, et al. 2023). Conversely, insufficient intake of vitamins A/D has been shown to suppress the production of antimicrobial proteins (e.g., lysozyme and α‐defensin 5), contributing to epithelial barrier compromise (Filipe Rosa et al. 2023). DMC group gene expression of *ZO‐1* and *Occludin* was markedly higher than that of the normal control, which may be attributed to these dietary nutrients. These results indicate that DM protects against DSS‐induced intestinal barrier damage by enhancing barrier function.

Intestinal barrier dysfunction induces pathogen translocation into deeper intestinal layers or distant organs, exacerbating oxidative stress and inflammatory response, which ultimately contributes to intestinal tissue injury. Nuclear factor‐kappaB (NF‐κB), a pivotal transcriptional regulator of pro‐inflammatory cascades, exhibits sustained activation in chronic immune disorders such as inflammatory bowel disease (IBD) and irritable bowel syndrome (IBS), driving pathological progression (Mukherjee et al. 2024). TLR4 recognizes and binds lipopolysaccharide (LPS), activating the MyD88‐dependent pathway to induce NF‐κB‐driven production of inflammatory mediators such as TNF‐α, IL‐1β, and IL‐6 (Bertani and Ruiz 2018; Byrd‐Leifer et al. 2001; Mukherjee et al. 2024). These proinflammatory mediators could compromise tight junction integrity, which intensifies inflammation and barrier impairment, establishing a perpetuating feedback loop (Kaminsky et al. 2021; X. Zhang et al. 2023). DSS upregulates TLR4 (a pattern recognition receptor) and activates the MyD88‐dependent pathway, triggering NF‐κB dimer release and p65 nuclear localization sequence (NLS) exposure, thereby inducing pro‐inflammatory mediator secretion (Yuan et al. 2023). Previous studies demonstrated that lysozyme specifically binds the endotoxin‐binding pocket of TLR4 through its N‐terminal antimicrobial peptide motif. This binding might reduce the expression of proinflammatory cytokines TNF‐α, IL‐6, and IL‐1β in murine macrophages stimulated by either lipopolysaccharide (LPS) or interferon‐γ (IFN‐γ) (Ibrahim et al. 2017). Moreover, lactoferrin, conjugated linoleic acid, and vitamin D mitigate inflammatory responses by suppressing the TLR4/MyD88/NF‐κB signaling pathway as confirmed in animal models of disease (H. Y. Li et al. 2021; Luo et al. 2023; Tang et al. 2020). Casein, α‐lactalbumin, and L‐isoleucine have been demonstrated to alleviate DSS‐induced colitis by suppressing the TLR4/MyD88/NF‐κB signaling pathway (Chu et al. 2023; Ma et al. 2024; Mao et al. 2022). The inhibitory effect of nutritional components in DM on the TLR4/MyD88/NF‐κB pathway likely underlies its ameliorative action against DSS‐induced colitis. Nevertheless, whether a single component in DM mediates the predominant inhibitory effect or multiple constituents confer synergistic inhibition remains subject to experimental verification.

The gut microbiota plays a critical role in the pathogenesis and progression of UC (Gu et al. 2022). Research indicates that gut microbiota dysbiosis compromises intestinal mucosal barrier integrity, facilitating pathogen invasion into tissues and enhancing bacterial adhesion to the epithelium, ultimately triggering acute or chronic inflammation (Y. Wang et al. 2022). The human gut microbiota is primarily composed of the Firmicutes and Bacteroidota, which collectively represent approximately 90% of the total microbial abundance (Illiano et al. 2020). Patients with UC commonly exhibit reduced gut microbiota richness and diversity, characterized by decreased abundance of the Firmicutes, Desulfobacterota, and Verrucomicrobiota, alongside increased Proteobacteria abundance (Zhu et al. 2022). The Firmicutes/Bacteroidetes (F/B) ratio plays a key role in gut microbiota homeostasis, with decreased ratios observed in inflammatory bowel disease (IBD) patients (Dou et al. 2020). It is well established that an increase in Proteobacteria is a hallmark of gut dysbiosis. DSS induction significantly elevated Bacteroidota abundance while reducing Firmicutes and Desulfobacterota levels, thereby decreasing the F/B ratio. DM treatment effectively counteracted these dysbiotic alterations. The genus *Bacteroides* (Bacteroidota) typically serves as a gut commensal, yet demonstrates significantly increased abundance in UC patients (Kang et al. 2023). Furthermore, studies indicate that Bacteroides‐derived proteins constitute 40%–60% of the microbiota‐sourced proteome positively correlated with UC disease activity (Mills et al. 2022). *Bacteroides fragilis* activates the TLR4/MyD88/NF‐κB signaling pathway, thereby triggering pro‐inflammatory responses (Z. Wu et al. 2024). *Bacteroides thetaiotaomicron* utilizes MUC2 glycans as an energy source, compromising intestinal barrier integrity (Yao et al. 2021). Moreover, the pathogenic taxa *norank_Muribaculaceae*, *Prevotellaceae_UCG‐001*, and *Eubacterium_fissicatena_group* potentiate pro‐inflammatory responses (Yalan Li et al. 2022; Yexun Zhou, Wei, et al. 2023; Yilin Zhou et al. 2024). These pathobionts colonize the intestines of DSS‐induced UC murine models, which might contribute to diarrheal manifestations and gastrointestinal dysfunction. Multiple Firmicutes taxa, particularly SCFA‐producing genera like *Lachnoclostridium*, exert protective effects that mitigate colorectal cancer risk and ameliorate colitis (Mo et al. 2024; W.‐Q. Zhang et al. 2022). Research has demonstrated that *Enterorhabdus* possesses both anti‐inflammatory and hepatoprotective properties. Beneficial increases in this genus have been reported in studies investigating the amelioration of 5‐fluorouracil‐induced intestinal damage (Huang et al. 2023). *Parvibacter* represents a potentially therapeutic genus that mediates beneficial effects in colitis management (Cheng et al. 2023; X. Wang et al. 2024). To assess functional changes during microbial structural shifts, we conducted PICRUSt‐based KEGG pathway prediction analysis (Langille et al. 2013). DM treatment upregulated pathways related to the metabolism of cofactors and vitamins, as well as amino acid metabolism, suggesting that DM may enhance metabolic functions in DSS‐induced colitis mice. Dysregulation of lipid metabolism and glycan biosynthesis/metabolism—particularly upregulation of pathways such as secondary bile acid biosynthesis and glycosaminoglycan degradation—may contribute to the progression of colitis (L. Chen et al. 2025; Lee et al. 2009). Previous studies revealed significant alterations in amino acid metabolism in both active inflammatory bowel disease (IBD) patients and IBD rat models, with elevated levels of colitis‐protective glycine (Xie et al. 2021). Furthermore, dysregulation of the pantothenate and CoA biosynthesis pathway was observed in colorectal cancer patients (Yi et al. 2023). Thus, DM may attenuate UC by modulating gut microbiota composition and functionality.

Collectively, this study demonstrates that DM significantly ameliorates clinical symptoms and reduces histopathological damage in the colon of UC model mice. The protective mechanisms involve: (1) enhanced expression of intestinal barrier function‐related genes, (2) suppression of TLR4/MyD88/NF‐κB pathway activation and pro‐inflammatory cytokine release, and (3) restoration of gut microbiota homeostasis. Current limitations in UC therapeutics highlight the need for safer, more effective treatments. Our findings provide a theoretical foundation for developing DM‐based interventions against intestinal inflammation. As a natural food matrix, DM exhibits potential for UC prevention and treatment through synergistic anti‐inflammatory effects and microbial modulation.

## Author Contributions

**Lin Yang:** conceptualization (equal), formal analysis (equal), formal analysis (equal), methodology (equal), methodology (equal), writing – original draft (equal), writing – original draft (equal). **Hua Ni:** conceptualization (equal), methodology (equal), writing – review and editing (equal). **Xiaogang Gou:** conceptualization (equal). **Bingqian Zhong:** supervision (equal). **Keyi Wen:** conceptualization (equal), investigation (equal). **Yingying Zhang:** conceptualization (equal). **Shicui Zhang:** conceptualization (equal), writing – review and editing (equal). **Yutao Wang:** conceptualization (equal), funding acquisition (equal), project administration (equal), supervision (equal), writing – review and editing (equal).

## Ethics Statement

This study was performed strictly per the Guidelines for the Care and Use of Laboratory Animals promulgated by the National Institutes of Health, and experimental protocols and animal husbandry practices received approval from Jiangsu Huachuang Xinnuo Pharmaceutical Technology Co. Ltd.'s IACUC (Approval code: SY‐2024‐05‐15001, approval date: 15 May 2024).

## Consent

The authors have nothing to report.

## Conflicts of Interest

The authors declare no conflicts of interest.

## Data Availability

The data supporting the findings of this study are available from the corresponding author upon reasonable request.
